# Oncobox Bioinformatical Platform for Selecting Potentially Effective Combinations of Target Cancer Drugs Using High-Throughput Gene Expression Data

**DOI:** 10.3390/cancers10100365

**Published:** 2018-09-29

**Authors:** Maxim Sorokin, Roman Kholodenko, Maria Suntsova, Galina Malakhova, Andrew Garazha, Irina Kholodenko, Elena Poddubskaya, Dmitriy Lantsov, Ivan Stilidi, Petr Arhiri, Andreyan Osipov, Anton Buzdin

**Affiliations:** 1 National Research Centre “Kurchatov Institute”, Centre for Convergence of Nano-, Bio-, Information and Cognitive Sciences and Technologies, 1 Akademika Kurchatova pl., Moscow 123182, Russia; 2 Laboratory of Clinical and Genomic Bioinformatics, I.M. Sechenov First Moscow State Medical University (Sechenov University), Moscow 119991, Russia; buzdin@oncobox.com; 3 OmicsWay Corp., Walnut, CA 91798, USA; garazha@oncobox.com; 4 Shemyakin-Ovchinnikov Institute of Bioorganic Chemistry, Moscow 117997, Russia; khol@mail.ru (R.K.); galina_vm@mail.ru (G.M.); 5 D. Rogachev Federal Research Center of Pediatric Hematology, Oncology and Immunology, 1 Samory Mashela str., Moscow 117997, Russia; suntsova86@mail.ru; 6 Orekhovich Institute of Biomedical Chemistry, Moscow 119832, Russia; irkhol@yandex.ru; 7 Center of Personalized Oncology, I.M. Sechenov First Moscow State Medical University (Sechenov University), Moscow 119991, Russia; podd-elena@ya.ru; 8 Clinical Center Vitamed, Moscow 121309, Russia; 9 Kaluga Regional Oncological Hospital, Kaluga 248007, Russia; lantsov@mail.ru; 10 N.N. Blokhin Russian Cancer Research Center, Moscow 115478, Russia; info@ponkc.com (I.S.); arhiri@mail.ru (P.A.); 11 State Research Center-Burnasyan Federal Medical Biophysical Center of Federal Medical Biological Agency, Moscow 123098, Russia; andreyan.osipov@gmail.com

**Keywords:** target therapeutics, tyrosine kinase inhibitors, rapalogues, cancer, synergistic combinations, drug resistant tumor cells, bioinformatics, molecular pathways

## Abstract

Sequential courses of anticancer target therapy lead to selection of drug-resistant cells, which results in continuous decrease of clinical response. Here we present a new approach for predicting effective combinations of target drugs, which act in a synergistic manner. Synergistic combinations of drugs may prevent or postpone acquired resistance, thus increasing treatment efficiency. We cultured human ovarian carcinoma SKOV-3 and neuroblastoma NGP-127 cancer cell lines in the presence of Tyrosine Kinase Inhibitors (Pazopanib, Sorafenib, and Sunitinib) and Rapalogues (Temsirolimus and Everolimus) for four months and obtained cell lines demonstrating increased drug resistance. We investigated gene expression profiles of intact and resistant cells by microarrays and analyzed alterations in 378 cancer-related signaling pathways using the bioinformatical platform Oncobox. This revealed numerous pathways linked with development of drug resistant phenotypes. Our approach is based on targeting proteins involved in as many as possible signaling pathways upregulated in resistant cells. We tested 13 combinations of drugs and/or selective inhibitors predicted by Oncobox and 10 random combinations. Synergy scores for Oncobox predictions were significantly higher than for randomly selected drug combinations. Thus, the proposed approach significantly outperforms random selection of drugs and can be adopted to enhance discovery of new synergistic combinations of anticancer target drugs.

## 1. Introduction

Emergence of target drugs significantly increased success rates for the therapy of many cancer types. For example, in renal cell carcinoma, which is not sensitive to radiation or generalized chemotherapy, the introduction into the clinical practice of a new class of target drugs—tyrosine kinase inhibitors (TKIs) increased the response rate of the therapy to the impressive value of up to 70% of patients [1]. Another successful example is the use of a TKI Pazopanib in ovarian cancer patients, which resulted in significant increase of progression-free survival [2]. Target therapeutics (anti-angiogenic and tyrosine kinase inhibitors) significantly improved treatment of differentiated thyroid carcinoma [3].

Multiple target drugs are currently under investigation in thousands of clinical trials. For example, novel Janus Kinase-2 inhibitor, Fedratinib, showed promising results in patients with myelofibrosis [4]. Despite significant advances in the treatment of cancer, many patients stop responding to target therapies after completion of several courses of treatment, which leads to relapse of the disease [3,5]. Apparently, this can be result of cancer cells adaptation to the drug(s) used followed by clonal expansion of the resistant cells [6]. The resistant cancer cells can promote survival through activation of alternative proliferative mechanisms, distinct from those inhibited by the respective target drugs [7]. Consequently, control over these molecular mechanisms may prevent tumor adaptation to the target therapeutics.

**Figure 1:**
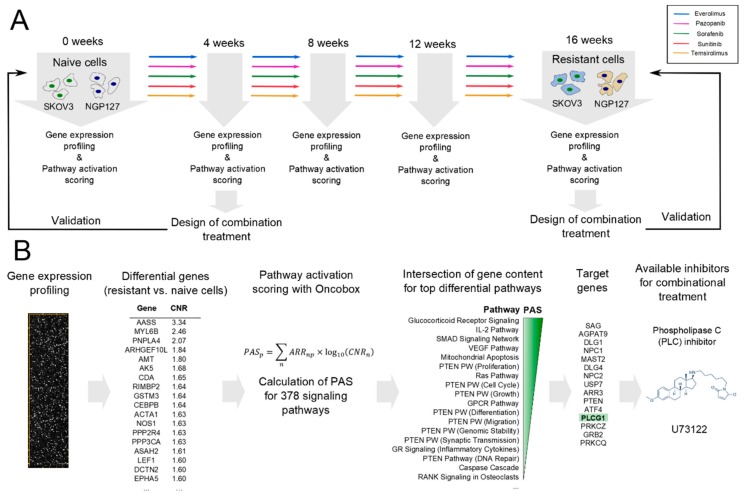
Overall design of the study. (**A**) Adaptation of cell lines to target drugs. (**B**) Bioinformatical pipeline for finding target drugs combinations.

Previously, we showed that long-term culturing of human ovarian carcinoma SKOV-3 and neuroblastoma NGP-127 cell lines with target drugs, such as TKIs Pazopanib, Sorafenib, Sunitinib, and mTOR inhibitors Everolimus and Temsirolimus significantly alters their sensitivity to radiation therapy [8]. This long-term culturing resulted in an increase of half maximal inhibitory concentration (IC_50_) of the corresponding drugs according to MTT test (Table 2 from Sorokin et al., 2018). Thus, after four months of culturing, most of the cell populations developed drug-resistant phenotypes. Every four weeks, using microarrays (CustomArray, Inc., Bothell, WA, USA) we profiled gene expression in aliquots of the cells cultured (Figure 1A). The gene expression data were deposited in the Gene Expression Omnibus (GEO) database under accession numbers GSE97750 and GSE97751.

Here we present an approach for selecting potentially effective combinations of target drugs using these high throughput gene expression data. The gene expression data were used to calculate activations of the intracellular signaling pathways using the bioinformatical platform Oncobox. Our approach postulates that the most effective targets for the inhibition with selective anticancer drugs are those that participate in the maximum number of upregulated signaling pathways in the drug-resistant cells. The enclosed pipeline produces a list of proteins, which could be selectively inhibited to prevent individual tumor resistance to target therapeutics. To experimentally investigate this approach, we tested 13 predicted and 10 random combinations of drugs and/or selective inhibitors. Three out of 13 predicted and one out of ten random combinations showed synergistic effect on cancer cell survival. Moreover, mean synergy score for predicted combinations were significantly higher than for randomly selected drugs/inhibitors combinations.

## 2. Results

### 2.1. Signaling Pathway Activation in Drug-Resistant Cell Lines

We analyzed alterations in the activities of intracellular signaling pathways using bioinformatical platform Oncobox (Figure 1B). Oncobox utilizes an algorithm that quantitatively analyzes the extents of molecular pathway activation by calculating the value of pathway activation strength (PAS) for each molecular pathway under investigation [9,10]. The PAS approach has proven its advantage over the level of single gene expressions by producing less significant batch effects and platform bias, as shown for many available microarray, deep sequencing, and quantitative proteomics platforms [11]. Positive PAS value indicates upregulation of a molecular pathway compared to the control biosample or group of biosamples, negative PAS value—downregulation, and zero value suggests no changes in pathway activation. The higher PAS value means higher pathway activation, and vice versa [12].

For each pair drug-cell line, at every timepoint across the study, we calculated PAS values for 378 intracellular signaling pathways previously associated with cancer development [13]. As the controls served as the reference for measuring pathways activation, we used a pool of control cells cultured without target drugs added (five biological replicates for each cell type). For all the experiments, PAS values are shown on Supplementary Table S1.

**Figure 2:**
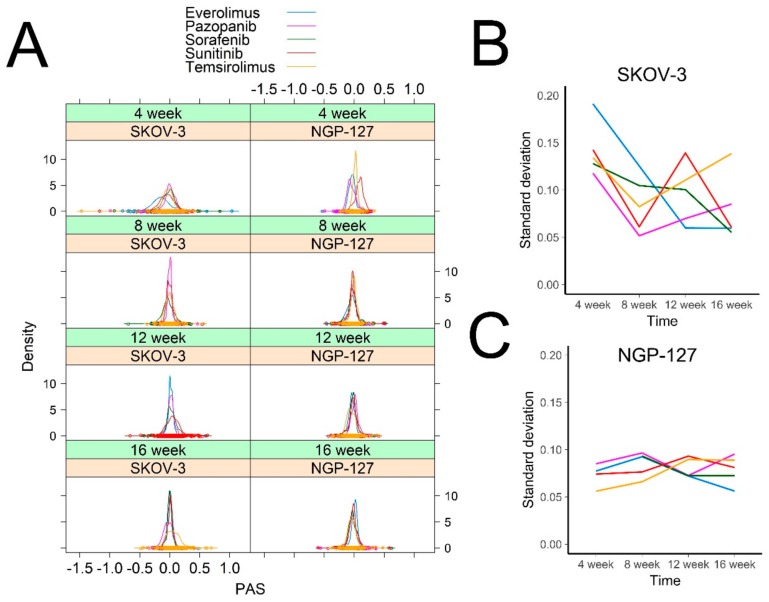
Distribution of PAS values over time. (**A**) A density plot was built for each cell line for each drug and for each timepoint. Each curve shows density of Pathway Activation Strength for 378 signaling pathways. Density plots were built using Lattice R package (**B**,**C**). Standard deviation of PAS values for SKOV-3 (**B**) and NGP-127 (**C**) cells treated with target drugs for 1 to 4 months.

The pathway activation profiles in response to target drugs were clearly different among the NGP-127 and SKOV-3 cells. Interestingly, at the initial 4-week point following culturing with the target drugs, in the SKOV-3 cells the molecular pathways showed at least two-fold bigger standard deviation in their activation levels (PAS) compared to the NGP-127 cells (Figure 2A,B). In SKOV-3, for most of the drugs this standard deviation significantly decreased after four months of culturing compared to the initial point (Figure 2A,B); in contrast, for the NGP-127 cells, this standard deviation remained stably low and relatively constant in all observations and timepoints (Figure 2C). Thus, the molecular mechanisms of acquiring drug resistance seem to be cell-type-specific and different in NGP-127 and SKOV-3 cells.

### 2.2. Prediction and Experimental Testing of Drugs Combinations

To get a deeper insight into the mechanisms of acquiring drug resistance in these cell types, we focused on the signaling pathways that were altered after exposure to target drugs. For each investigated timepoint, we selected ten percent of the most strongly affected pathways and then intersected lists of gene products, which formed these pathways. We hypothesized that targeting proteins corresponding to the most frequently occurring intersected gene products may be beneficial to suppress acquisition of drug resistance. The targets predicted at 4-week time point were used to select combinations in naïve cells, and those predicted at a 16-week time point were used to select combinations in adapted cells. The predicted molecular targets varied significantly across drugs, cell lines, and timepoints investigated (Table 1).

**Figure 3:**
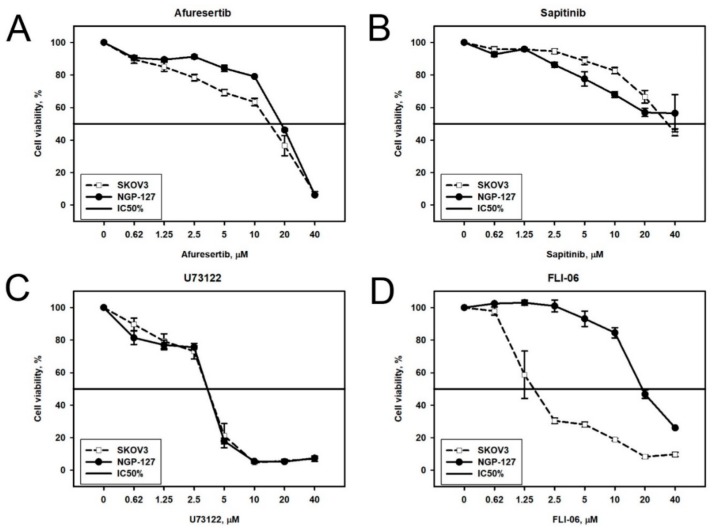
Viability of SKOV-3 and NGP-127 cells treated with different concentrations of target drugs: (**A**) Akt inhibitor Afuresertib; (**B**) EGFR and ErbB inhibitor Sapitinib; (**C**) phospholipase C inhibitor U73122; and (**D**) Notch inhibitor FLI-06. Viability and IC_50_ were measured with MTT (3-[4,5-dimethylthiazol-2-yl]-2,5-diphenyltetrazolium bromide) test.

We next attempted to experimentally investigate if inhibition of the predicted molecular targets may be effective for eliminating drug resistance in the corresponding cell types. To do this, we used nine target drugs/inhibitors for twenty predicted molecular targets (Table 2). Prior to combinational cytotoxicity experiments, we examined cytotoxicity of these drugs/inhibitors alone on the NGP-127 and SKOV-3 cells for the selection of optimal concentrations to use with the baseline target drugs (Table 3). For most of the components tested, there was no significant difference in the half-inhibitory concentration (IC_50_) on both cell lines (Figure 3A–C). However, the specific inhibitor of a transmembrane signaling protein Notch, FLI-06, had markedly lower IC_50_ for SKOV-3 cells than for the NGP-127 cells (Figure 3D). This suggests that Notch signaling may have a crucial role in the survival of SKOV-3 cells and that this effect is cell type specific.

We next exposed the intact (naïve) SKOV-3 and NGP-127 cells to combinations of the drugs/inhibitors. Each such combination contained one initial drug (Pazopanib, Sorafenib, Sunitinib, Everolimus, or Temsirolimus) and another drug or inhibitor, which was predicted to complement activity of the former.

**Figure 4:**
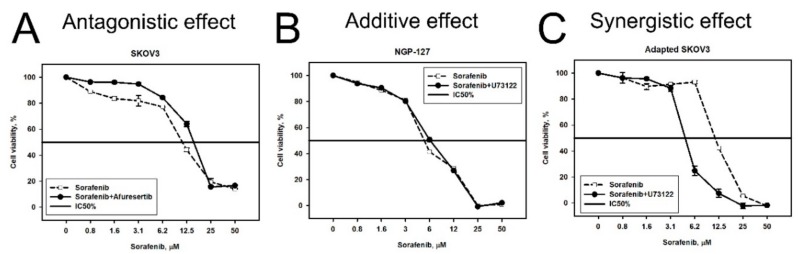
Examples of antagonistic (**A**), additive (**B**), and synergistic (**C**) effect of inhibitor/drug combinations. Dashed line corresponds to single drug, solid line—combination. Survival of cells, subjected to combination of drugs was normalized by viability of cells, treated with the drug used in constant concentration. For example, the viability of SKOV-3 cells subjected to Sorafenib and Afuresertib was divided by viability of SKOV-3 cells subjected to IC_20_ of Afuresertib alone.

Design of the experiment comprised the addition to the cell culturing medium of a predicted drug/inhibitor at the constant concentration corresponding to its IC_20_, while concentrations of the initial drugs were variable in order to measure their IC_50_ parameters in the presence of an additional predicted drug/inhibitor. As before, the MTT test was used to investigate cell viability under all the conditions. Cytotoxicity curves on the viability graphs reflect the effects of titration of the initial target drug with the addition of a constant inhibitor concentration (Figure 4). Such curves were built using the control baseline for the intact cells grown in the presence of IC_20_ of the corresponding predicted drug/inhibitor alone. We estimated effect of a combination by comparing viability of cells subjected to the combination (Figure 4, solid lines) with viability of cells subjected to initial single agent (Figure 4, dashed lines). Synergy was assessed using the Bliss independence model (see Materials and Methods section). We used Bliss independence criterion, because it is applicable to our experimental data, where a dose-response curve is measured in the presence/absence of another drug in a single concentration [14]. We considered an effect of combination synergistic if the Bliss score was more than 5 (Figure 4C); the effect was considered antagonistic if the Bliss score was less than −5 (Figure 4A). The effect of combination was considered additive in other cases (Figure 4B).

**Figure 5:**
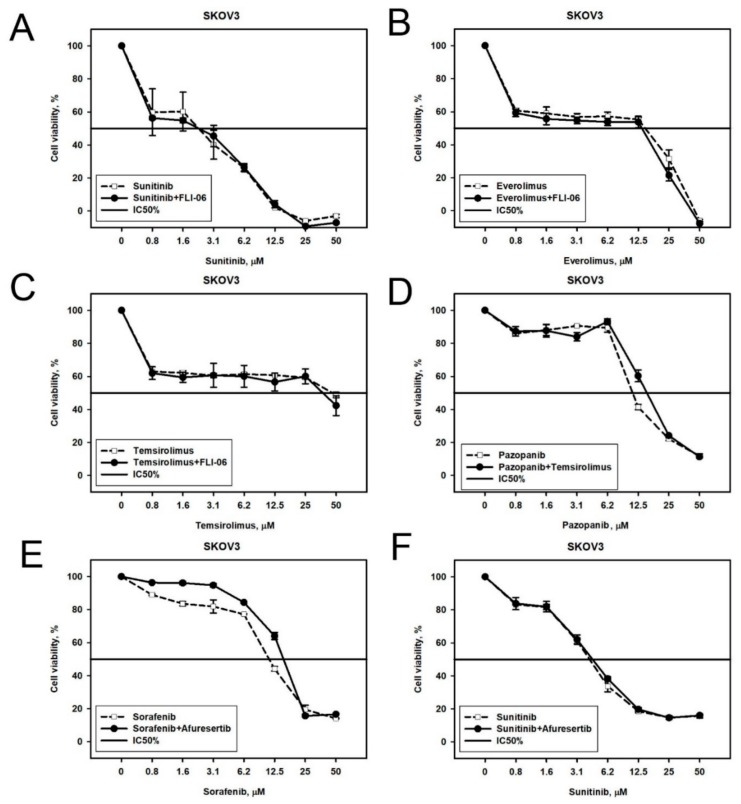
Viability of naïve SKOV-3 cells treated with different combinations of target drugs: (**A**–**C**) Sunitinib, Everolimus, or Temsirolimus, respectively, in combination with Notch inhibitor FLI-06 or alone; (**D**) Pazopanib in combination with mTOR inhibitor Temsirolimus; (**E**,**F**) Sorafenib or Sunitinib, respectively, in combination with Akt inhibitor Afuresertib or alone. Dashed line corresponds to single drug, solid line—combination.

FLI-06 in SKOV-3 cells. Notch protein, molecular target of FLI-06, was predicted to be a potent drug resistance actor for the SKOV-3 cells cultured with Sunitinib, Everolimus, or Temsirolimus. When we tested FLI-06 in combination with Sunitinib, Everolimus, or Temsirolimus, we observed its additive effect of FLI-06 with the above drugs (Figure 5A–C). Interestingly, FLI-06 also showed additive effects for the combinations with Sorafenib and Pazopanib (Figure S1A,B), even though these combinations were not predicted by the Oncobox platform. Together, these findings point to an important role of Notch signaling in SKOV-3 cells drug resistance. However, Notch signaling is unlikely involved in acquiring TKI resistance in SKOV-3 cells, because combinations of TKIs and FLI-06 acted additively in these cells, independently from Oncobox predictions.

Afuresertib in SKOV-3 cells. Akt protein, the molecular target of a drug Afuresertib, was predicted as a potential specific target in SKOV-3 cells treated with Sorafenib or Sunitinib for four weeks. However, we could only observe the additive effect in combination with Sunitinib (Figure 5F), but not with Sorafenib, where the effect was antagonistic (Figure 5E).

Temsirolimus in SKOV-3 cells. The downstream target of Akt signaling—mTOR—was over-represented in the signaling pathways, which were upregulated in SKOV-3 cells following four weeks of treatment with Pazopanib. In good agreement, we observed additive effects of mTOR inhibitor drug Temsirolimus with Pazopanib in SKOV-3 cells (Figure 5D).

**Figure 6:**
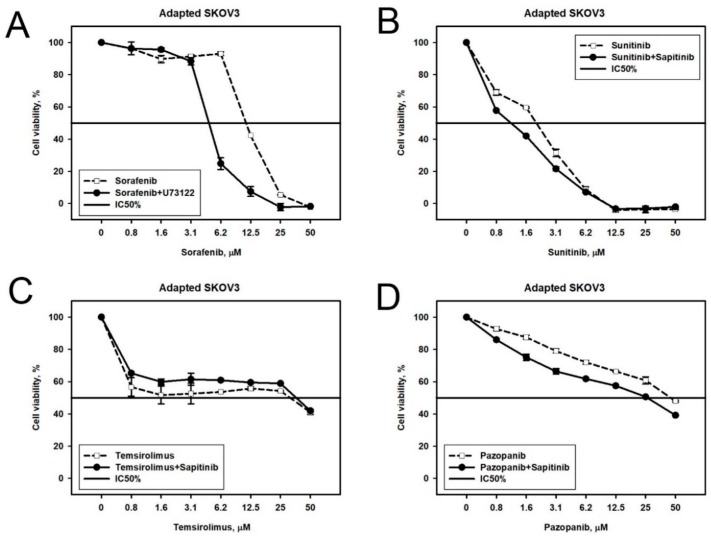
Viability of target drug resistant SKOV-3 cells treated with different combinations of target drugs: (**A**) Sorafenib-resistant cells treated with (i) combination of Phospholipase C inhibitor U73122 and Sorafenib or (ii) Sorafenib alone; (**B**) Sunitinib-resistant cells treated with (i) combination of *EGFR* inhibitor Sapitinib and Sunitinib or (ii) Sunitinib alone; (**C**) Temsirolimus-resistant cells treated with (i) combination of Temsirolimus and EGFR inhibitor Sapitinib or (ii) Temsirolimus alone; (**D**) Pazopanib-resistant cells treated with (i) combination of Pazopanib and EGFR inhibitor Sapitinib or (ii) Pazopanib alone. Dashed line corresponds to single drug, solid line—combination.

U73122 in SKOV-3 cells. According to the Oncobox combination prediction, we next tested Phospholipase C inhibitor U73122 in combination with Sorafenib in Sorafenib-resistant SKOV3 cells. We observed synergistic cytotoxicity of U73122 and Sorafenib (Figure 6A). We also tested the same combination on the naïve SKOV-3 cells and again observed a synergistic effect (Figure S1D), although not predicted by the Oncobox platform.

Sapitinib in SKOV-3 cells. We next tested predicted combinations of Sapitinib, inhibitor of ErbB2, ErbB3, and EGFR, in Pazopanib-, Sunitinib-, and Temsirolimus-resistant SKOV-3 cell populations. We found that these drugs could act synergistically in case of Sunitinib or Pazopanib (Figure 6B,D), but the effect was not synergistic in case of Temsirolimus (Figure 6C).

**Figure 7:**
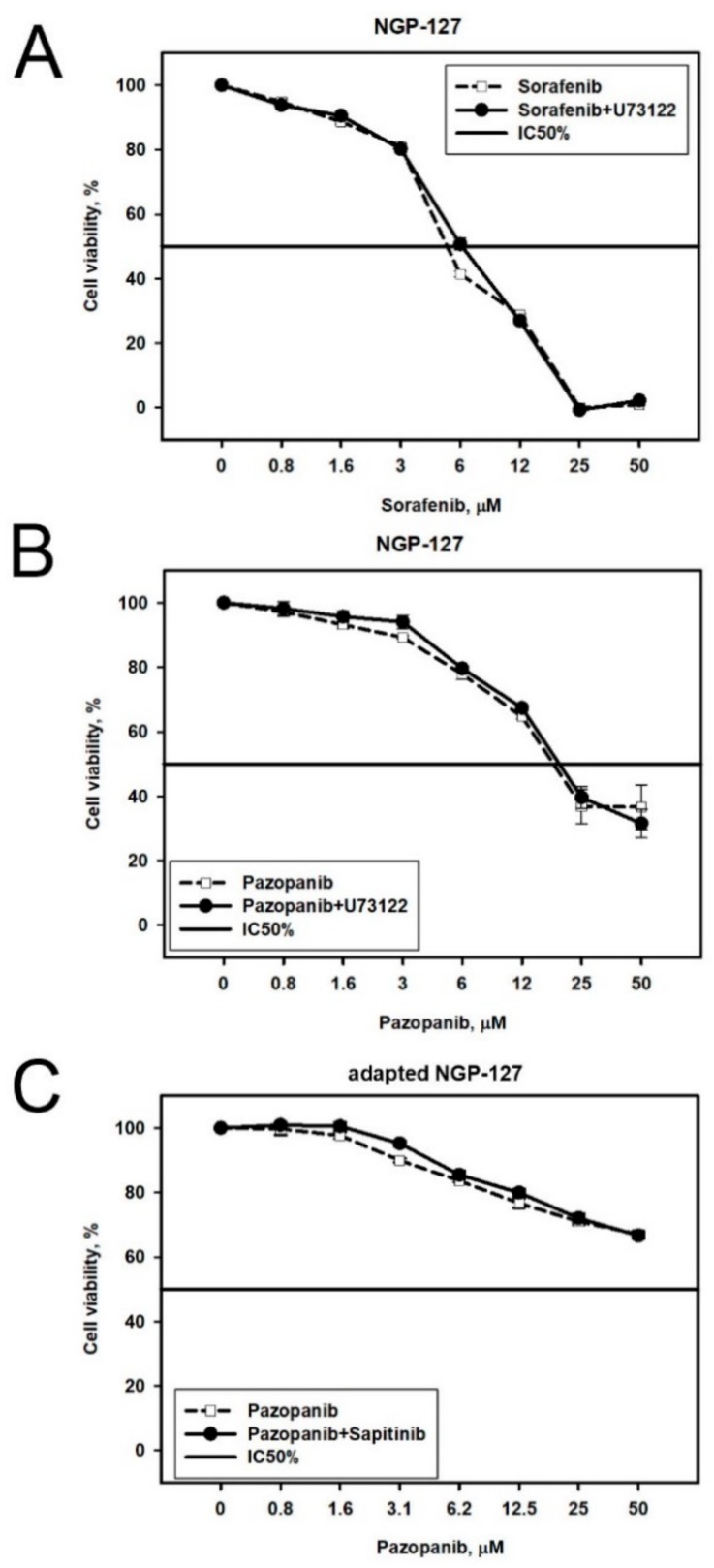
Viability of NGP-127 cells treated with different combinations of target drugs: (**A**) Sorafenib with Phospholipase C inhibitor U73122; (**B**) Pazopanib with Phospholipase C inhibitor U73122; and (**C**) NGP-127 cell lines adapted to Pazopanib and treated with (i) combination of Pazopanib and EGFR inhibitor Sapitinib or (ii) Pazopanib alone. Dashed line corresponds to single drug, solid line—combination.

U73122 and Sapitinib in NGP-127 cells. We examined three Oncobox predictions made for NGP-127 cell lines. On the naïve NGP-127 cells, the combinations of Sorafenib + U73122 and Pazopanib + U73122 worked additively (Figure 7A,B), and the same was observed for the combination of Pazopanib + Sapitinib on the Pazopanib-resistant cell lines (Figure 7C).

Random drugs/inhibitors combinations. We also tested 10 random drugs/inhibitors combinations, which were not predicted by our method. Of those, one combination showed synergistic effect, in five cases this effect was additive and in four cases it was antagonistic (Figure S1). However, the synergistic effect was seen in the naïve SKOV-3 cells treated with Sorafenib and U73122, which was predicted to be effective in the Sorafenib-resistant SKOV-3 cells.

Conclusion. Overall, we investigated 13 predictions of the drugs/inhibitors combinations done by the Oncobox platform. In three cases we observed a synergistic effect of the drugs and in eight cases the effect of the drug combination was additive. The antagonistic effect was seen for only two combinations. The difference between Bliss synergy scores calculated for Oncobox predicted combinations and scores for random combinations was significant according to *t*-test (*p*-value < 0.05, Table S2). Thus, our method identifies combinations that have a synergistic effect higher than randomly selected combinations. Taken together, these findings suggest that the Oncobox method outperforms random selection in predicting potentially effective combinations of target drugs.

## 3. Discussion

The quantitative pathway activation analysis, which was used here for the selection of drug combinations, has already shown to be effective for finding new biomarkers of cancer and other human diseases [10,15,16,17]. The analytical approach presented here is based solely on the intersection of the most strongly upregulated signaling pathways, and the selection of drugs/inhibitors which targeted the maximum number of these pathways.

Previously, we successfully used this approach for a single case of human acute myeloid leukemia with *AML1-ETO* fused oncoprotein [13,18]. However, it remained unclear whether the same method will be reproducibly effective for the other objects. It was also unexplored if this approach provides advantage compared to randomly taken combinations of drugs/inhibitors.

Here, we examined five target drugs on two different cell lines for a period of up to four months. Somewhat similar experiments which focused on the discovery of synergistic drug combinations were conducted by Di Nicolantonio and coauthors [19]. However, in that study the cell lines were grown in the presence of chemotherapeutic drugs for only six days, which is poorly correlated with the duration of chemotherapy in clinical practice. Several other related attempts were recently published, but they had either a lower number of cell populations analyzed [20], or lower number of drugs tested [21], or both [22]. Other computational approaches for predicting synergistic pairs of drugs using gene expression data were also reported. He et al. described a personalized predictor of drug combinations for leukemia patients which was successfully validated in 10 out of 24 cases [23]. The Drug-Induced Genomic Residual Effect (DIGRE) model also showed promising results in predicting effective pairs of drugs; however it was only validated for a single combination: gefitinib and docetaxel in various concentrations [24]. The Ranking-system of Anticancer Synergy (RACS) is another transcriptomic-based approach for selecting effective drug combinations [25]. Approximately 60% of RACS-predicted combinations were shown to act synergistically, while 13% of randomly selected pairs showed same effect. Integrative pharmacogenomic approach for predicting effective combinations was also proposed [26]. The authors experimentally tested only one combination of drugs, which appeared to act in a synergistic manner. Interestingly, the DREAM consortium assessed performance of 32 previously reported methods for predicting synergistic combinations in B cell lymphoma and only four of them were significantly better than random guessing [27]. However, none of the above discussed studies reported synergistic effects in cells, which are already resistant to target drugs. Moreover, Oncobox provides a list of proteins, which should be targeted to overcome or prevent drug resistance. Thus, our approach may be used not only for repurposing of the existing drugs, but also for discovery of the novel target therapeutics.

**Figure 8:**
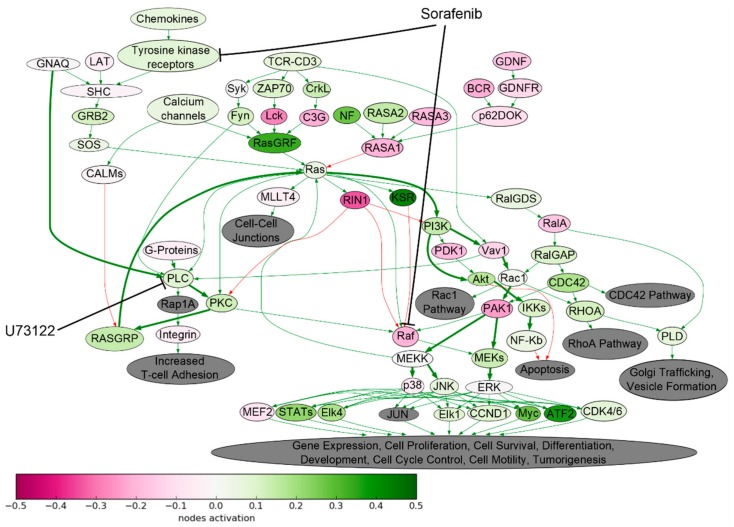
Ras pathway was hyperactivated in Sorafenib-resistant SKOV-3 cells. The pathway was visualized using Oncobox software. The pathway is shown as an interacting network, where green arrows indicate activation and red arrows indicate inhibition. Color depth of each node of the network corresponds to the logarithms of the case-to-normal (CNR) expression rate for each node, where “normal” is a geometric average between intact SKOV3 cells, the scale represents extent of up/downregulation. The molecular targets of Sorafenib and U73122 are shown by black arrows. Predicted bypass of nodes targeted by Sorafenib is shown on bold arrows.

Drug resistance acquisition by the tumor cells may be linked with the abnormal activation of signaling networks, which was not directly targeted by the drug. Our study revealed a highly effective synergistically acting combination of TKI—Sorafenib and Phospholipase C inhibitor—U73122. We found that Ras signaling pathway was significantly upregulated in Sorafenib-resistant SKOV-3 cells (Figure 8). Although Sorafenib targets tyrosine kinases and Raf proteins, thus inhibiting proliferation, the proliferation still can be induced via alternative branch of the same signaling pathway, by Ras-PI3K axis, which is not targeted by Sorafenib. The Ras-PI3K axis is activated by the Protein Kinase C, which, in turn, is a downstream target of Phospholipase C (Figure 8). Indeed, our study showed that Sorafenib and U73122 can act synergistically in inhibiting growth of human ovarian carcinoma SKOV-3 cells.

The Bliss synergy score for combination of U73122 and Sorafenib was the highest across this study. Unfortunately, U73122 was not yet tested in clinical trials, thus we are unable to estimate clinical feasibility of such combination. Another synergistic combination revealed was Pazopanib and Sapitinib. Pazopanib is already FDA approved for treatment of renal cancer, and concentration used in this study (0.8–50 µM) is lower than C_max_ = 58.1 μg/mL (equivalent to 132 μM) for 800 mg bid. Median time to achieve peak concentration is 2 to 4 h after the dose (data from FDA website). Sapitinib is undergoing clinical trials for several cancer types. In this study we used an 11 µM Sapitinib concentration (equivalent to 5.21 µg/mL). At the same time, previous clinical trials have indicated that C_max_ for this drug was 1.53 µg/mL for 300 mg bid and that peak concentration could be observed 1 to 2 h after administering the dose [28]. Sapitinib dosage, which is required to achieve C_max_ of at least 5 µg/mL, was not previously tested in clinical trials. However, lower concentrations of Sapitinib could possibly act in a synergistic manner with Pazopanib. The latter could be tested in further preclinical trials, which are needed to determine clinical feasibility of this combination.

Bliss synergy scores for drug combinations predicted by Oncobox were significantly higher than for the randomly selected drug combinations. Thus, the proposed method significantly outperforms random selection. However, standard deviation of synergy scores was high for some combinations. Thus, additional studies using other cell lines and/or initial drugs might be required.

Apparently, most of the predicted combinations failed to show synergism. This may be due to the fact that the differential genes previously identified from drug-resistant cell models may largely represent drug-induced transcriptional changes, rather than active resistant mechanisms. Nevertheless, this approach could potentially be used to find synergistic combinations in cell line experiments. These combinations should be further tested in clinical trials. In addition, such an approach can be used to identify personalized combinations of target drugs in cases, when both pre- and post-treatment material is available, e.g., after neoadjuvant therapy. However, drug combinations acting synergistically in cell lines may cause severe side effects when administered to patient. Thus, further trials are needed in order to investigate such critical issues as required dosage of drugs supplemented in combination and their possible side effects.

## 4. Materials and Methods

### 4.1. Biosamples

We used two human cell lines to profile responses to anticancer drugs. Cell lines were cultured as described previously [8]. Briefly, the NGP-127 and SKOV-3 cells were cultured on Dulbecco’s modified Eagle’s medium (DMEM; Gibco, Waltham, MA, USA) supplemented with 10% heat-inactivated fetal bovine serum (HyClone, Pittsburgh, PA, USA), 100 mkg/mL penicillin (Sigma, St. Louis, MO, USA), 100 U/mL streptomycin (Sigma, St. Louis, MO, USA), and 2mM *L*-glutamine (Sigma, St. Louis, MO, USA) at 37 °C and 5% CO_2_. The cells were grown in 25 cm^2^ or 75 cm^2^ flask (Greiner, Frickenhausen, Germany) and passaged for every 72 h. In order to obtain drug-resistant cell lines the cells were exposed to Sorafenib, Sunitinib, Pazopanib, Temsirolimus, or Everolimus for 16 weeks to obtain drug-resistant cell lines. The cells were washed three times a week and each time new media was added. The media was also supplemented with drug in initial concentration. Thus, concentration of the drug in the media was constant during 16 weeks of culturing. Drug combinations were further tested in MTT-test (described below). Together with culturing of cells with target drugs, we cultured intact SKOV-3 and NGP-127 cells in drug-free media for 4 months. We profiled gene expression in aliquots of control cells every four weeks and thus obtained five gene expression profiles for each cell line (0, 4, 8, 12, and 16 weeks). We pooled the control samples for pathway activation quantification: each experimental gene expression profile was normalized by geometric mean of corresponding control samples.

### 4.2. Cell Culturing and Viability Assay

For cell viability assay, we used intact cell lines and cell populations resistant to anticancer drugs. Cell viability was determined as described previously [8]. Briefly, it was evaluated by using the MTT (3-[4,5-dimethylthiazol-2-yl]-2,5-diphenyltetrazolium bromide) test [29]. The following drugs were tested (purchased at Selleckchem, Houston, TX, USA): Pazopanib, Sunitinib, Sorafenib, Everolimus, and Temsirolimus. For every cell line, the drugs were tested in the following concentrations: 0, 0.8, 1.56, 3.1, 6.25, 12.5, 25, and 50 µM. All the experiments were made in quadruplicate. After addition of the testing components, the plates were incubated for 72 h and then plates were centrifuged at 300 *g* for 10 min, followed by the removal of supernatant. Thirty microliters of 0.5 mg/mL solution of MTT (Sigma, St. Louis, MO, USA) was added to each well, and the plates were incubated for 2 to 4 h, then 100 µL of DMSO was added to each well for formazan crystals dissolving. The optical densities (OD) at 540 nm were measured using a plate reader Multiscan FC (ThermoScientific, Waltham, MA, USA). Cell viability was calculated using the formula: (OD treated cells − OD blank)/(OD control cells − OD blank) × 100%, where OD blank means OD in control wells containing no cells. IC_50_ values were deduced from dose–response curves using SigmaPlot software (Systat Software Inc., San Jose, CA, USA). IC_50_ values are given in Table 3. To quantify synergy, we calculated Bliss model scores using R CalculateSynergy function without baseline correction from SynergyFinder package [30]. This package utilizes the following model equation.

Y_BLISS_ = Y_1_ + Y_2_ − Y_1_Y_2_

The effect was considered synergistic if the corresponding Bliss score was more than 5, antagonistic—if less than −5, and additive if otherwise. The difference between Bliss scores for Oncobox predicted combinations and Bliss scores for random combinations was estimated using R *t*-test with default parameters.

### 4.3. Data Analysis

Gene expression profiles were deposited to GEO database with the accession numbers GSE97750 and GSE97751. Gene expression was quantile normalized using R preprocessCore package [31]. Extents of signaling pathway activation were assessed with the bioinformatical platform Oncobox. Output of the Oncobox algorithm is a list of Pathway Activation Scores (PAS). The formula used to calculate the pathway activation strength (PAS) for a given sample and a given pathway *p* is as follows.

$$PAS_{p}=\underset{n}{\sum }ARR_{np}\times \log_{10}\left(CNR_{n}\right)$$

Here the case-to-normal ratio, *CNR_n_*, is the ratio of expression levels for a gene *n* in the sample under investigation to the same average value for the control group of samples. *ARR* (activator/repressor role) equals to the following fixed values: −1, when the gene product *n* is a repressor of pathway excitation; 1, if the gene product *n* is an activator of pathway excitation; 0, when the gene product *n* can be both an activator and a repressor of the pathway; 0.5 and −0.5, respectively, if the gene product *n* is rather an activator or repressor of the signaling pathway *p*, respectively.

PAS can take both positive and negative values meaning over- or underactivation relative to control (untreated) samples. In total we calculated activation level of 378 intracellular signaling pathways. The signaling pathways knowledge base developed by SABiosciences [32] was used to determine structures of intracellular pathways. The list of signaling pathways investigated is presented in Supplementary Table S1. We selected ten percent of pathways with highest PAS and intersected lists of gene products which form those pathways. Genes that were presented in the maximum number of pathways were regarded as the potential candidates for targeting in combinational tests. The low molecular mass inhibitors were purchased at Selleckchem (Houston, TX, USA) and tested on the cell cultures in combination with the initial target drugs.

## 5. Conclusions

Here we report a method for predicting effective combinations of target drugs, which outperforms random guessing in selecting synergistic or at least additive combinations. The method is based on gene expression profiling of drug-resistant cell lines and subsequent pathway analysis with Oncobox software. Our algorithm provides a list of potential targets, which are involved in most pathways activated during acquiring of drug resistance. Synergy scores for Oncobox predicted combinations were significantly higher than for random combinations.

## Figures and Tables

**Table 1:** Target proteins potentially linked with acquisition of drug resistance at different timepoints. The experimentally investigated combinations are shown in bold.

Cell Line	Drug	4–8 Week	16 Weeks
**NGP-127**	**Sorafenib**	**Phospholipase C**, JNK1-2-3/MAP2K4-7	JAK1, JAK3
	Sunitinib	JNK1-2-3/MAP2K4-7	RAS, PI3K
	**Pazopanib**	**Phospholipase C**, Adenylate cyclase	**EGFR**
	Temsirolimus	JNK1-2-3/MAP2K4-7	RAS
	Everolimus	PRKACA	PI3K
**SKOV-3**	**Sorafenib**	**Akt**	**Phospholipase C**
	**Sunitinib**	**Notch, Akt**	**EGFR, ErbB2**, ADCYs
	**Pazopanib**	**mTOR**	**ErbB3**
	**Temsirolimus**	**Notch**	**EGFR-ErbB2**
	**Everolimus**	**Notch**	MAP2K6-MAP2K3

**Table 2:** Drugs/inhibitors used in this study and their molecular targets.

Drug/Inhibitor	Molecular Target
Temsirolimus	mTOR, FKBP12
Everolimus	mTOR, FKBP12
Sunitinib	VEGFR2 (Flk-1) and PDGFRβ
Sorafenib	Raf-1, B-Raf and VEGFR-2
Pazopanib	VEGFR1, VEGFR2, VEGFR3, PDGFR, FGFR, c-Kit and c-Fms
Afuresertib (GSK2110183)	Akt
Sapitinib (AZD8931)	EGFR, ErbB2 and ErbB3
FLI-06	Notch
U73122	Phospholipase C (PLC)

**Table 3:** Inhibitory concentrations of drugs/inhibitors used in this study.

Drug	SKOV-3		NGP-127	
	IC_20_ (µM)	IC_50_ (µM)	IC_20_ (µM)	IC_50_ (µM)
Afuresertib	2.7	17	8	19
FLI-06	1.5	2	11	20
U73122	3	3.2	1	3.5
Sapitinib	11	37	4.2	≥40
Sorafenib *		9.6		5.5
Pazopanib *		≥50		12
Sunitinib *		3		3.1
Temsirolimus *		17		11.8
Everolimus *		17.6		15.5

* Data taken from a past paper [8].

## Supplementary Materials

The following are available online at http://www.mdpi.com/2072-6694/10/10/365/s1, Table S1: Pathway Activation Scores for NGP-127 and SKOV-3 cells incubated with target drugs for various timepoints, Table S2: Dashed lines correspond to single drug, solid lines—combination, Figure S1: Viability of naïve SKOV-3 (A–F) or NGP-127 (G–J) cells treated with different combinations of target drugs. Bliss synergy scores are shown in brackets. (A) Pazopanib in combination with FLI-06 (−3.00); (B) Sorafenib in combination with FLI-06 (−0.29); (C) Pazopanib in combination with Sapitinib (2.43); (D) Sorafenib in combination with U73122 (5.86); (E) Sorafenib in combination with Temsirolimus (−0.29); (F) Sorafenib in combination with Everolimus (−4.71); (G) Pazopanib in combination with Everolimus (−10.36); (H) Pazopanib in combination with Temsirolimus (−4.43); (I) Sorafenib in combination with Everolimus (−9.46); and (J) Sorafenib in combination with Temsirolimus (−8.89).
