# Supplementary figures and images for: Oncobox Bioinformatical Platform for Selecting Potentially Effective Combinations of Target Cancer Drugs Using High-Throughput Gene Expression Data
Source: Cancers (Basel). 2018 Sep 29;10(10):365. doi: 10.3390/cancers10100365 (PMC6209915; doi:10.3390/cancers10100365)

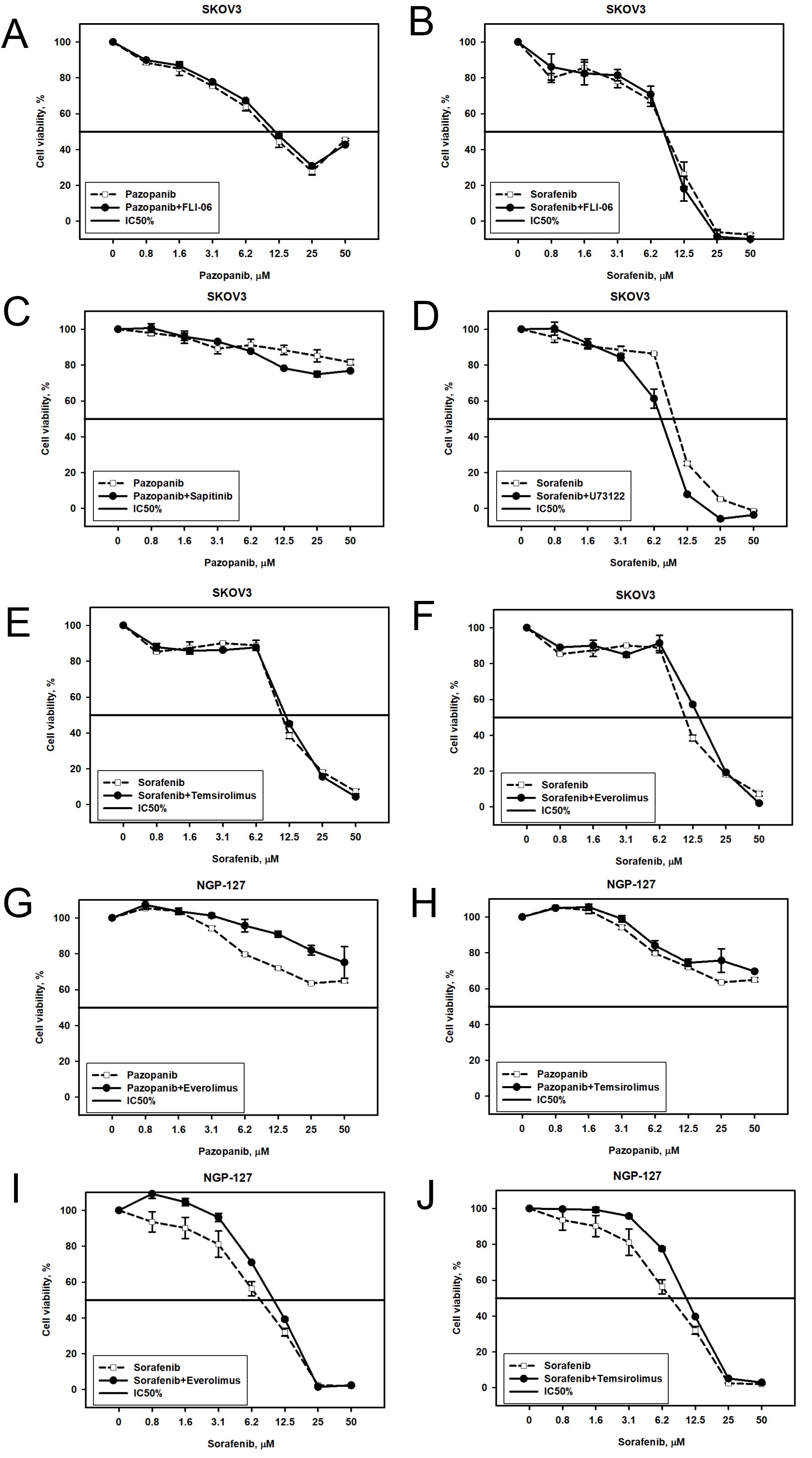

Supplement: Supplementary file 1 [file cancers-10-00365-s001.zip › cancers-356652-sup/Figure_S1.jpg]
